# Mouse avatar models of esophageal squamous cell carcinoma proved the potential for EGFR-TKI afatinib and uncovered Src family kinases involved in acquired resistance

**DOI:** 10.1186/s13045-018-0651-z

**Published:** 2018-08-29

**Authors:** Zhentao Liu, Zuhua Chen, Jingyuan Wang, Mengqi Zhang, Zhongwu Li, Shubin Wang, Bin Dong, Cheng Zhang, Jing Gao, Lin Shen

**Affiliations:** 1 Key laboratory of Carcinogenesis and Translational Research (Ministry of Education/Beijing), Department of Gastrointestinal Oncology, Peking University Cancer Hospital and Institute, 52 Fucheng Road, Haidian District, Beijing, 100142 China; 2 Key Laboratory of Carcinogenesis and Translational Research (Ministry of Education/Beijing), Department of Pathology, Peking University Cancer Hospital and Institute, 52 Fucheng Road, Haidian District, Beijing, 100142 China; 3 Department of Oncology, Peking University Shenzhen Hospital, 1120 Lianhua Road, Shenzhen, 518036 Guangdong China

**Keywords:** ESCC, Mouse avatar, Afatinib, Src family kinases, Acquired resistance

## Abstract

**Background:**

No approved targeted agents are available for esophageal squamous cell carcinoma (ESCC). Informative genomic analysis and mouse patient-derived xenografts (PDX) also called mouse avatar can greatly expedite drug discovery.

**Methods:**

Six ESCC cell lines and 7 out of 25 PDX models derived from 188 biopsies with clear molecular features were employed to evaluate the sensitivity of several EGFR blockers in vitro and in vivo, as well as the underlying antitumor mechanisms of the most promising EGFR-TKI afatinib. Mechanisms involved in acquired resistance of afatinib were explored based on established resistant cell lines and PDX models followed by an attempt to reverse resistance.

**Results:**

Compared with other EGFR blockers, the second-generation EGFR-TKI afatinib exerted superior antitumor effects in ESCC, and *EGFR* copy number gain (CNG) or overexpression was proposed to be predictive biomarkers. Afatinib played its antitumor effects by inhibiting EGFR downstream pathways, as well as inducing apoptosis and cell cycle arrest at G1. It was increased phosphorylation of Src family kinases (SFKs), rather than MET upregulation, that conferred to acquired resistance of afatinib. Dual blockade of EGFR and SFKs could overcome afatinib resistance and warrants validation in clinical practice.

**Conclusion:**

Both ESCC cell lines and PDXs with *EGFR* CNG or overexpression are potential candidates for afatinib, and concomitant EGFR/SFKs inhibition could reverse afatinib-acquired resistance caused by SFKs activation in ESCC.

**Electronic supplementary material:**

The online version of this article (10.1186/s13045-018-0651-z) contains supplementary material, which is available to authorized users.

## Background

Different from western countries, esophageal squamous cell carcinoma (ESCC) is the predominant histopathological type in China, which poses great threats to people’s health [1]. A large portion of patients with ESCC are diagnosed with advanced-stage disease and lose the opportunity for radical therapy, resulting in a very poor overall survival [2]. Current treatments for patients with unresectable advanced disease focus on chemotherapy and chemoradiotherapy, but the efficacy is quite limited [2, 3]. Although targeted therapies play an increasingly important role in the treatment of many cancers, no targeted agents are available in ESCC [4]. Consequently, developing new targeted agents based on potential targets is an urgent need in ESCC.

Currently, several large-scale genomics studies on ESCC have highlighted the roles of multiple recurrently dysregulated pathways and genes in ESCC, including receptor tyrosine kinase (RTK), cell cycle, Wnt/Notch, and Hippo pathways [5–7]. Among these altered pathways and genes, epidermal growth factor receptor (EGFR) is very promising, and attempts to target EGFR are never given up in ESCC even if no targeted agents are approved till now. Although *EGFR* mutation is rare in ESCC, frequency of *EGFR* amplification or copy number variation (CNV) ranges from 6 to 24.3% [5–9], suggesting a potential for EGFR-targeted therapy in ESCC.

EGFR blockers contain monoclonal antibodies (mAbs) and small-molecule tyrosine kinase inhibitors (TKI) and have been updated a few generations [10]. Despite the failure of some EGFR blockers in ESCC such as EGFR monoclonal antibodies (mAbs) cetuximab and tyrosine kinase inhibitors (TKIs) gefitinib as confirmed by several clinical trials [11–14], subsequent stratified analysis of these trials suggested that patients with squamous histopathological type, *EGFR* copy number gain (CNG), or overexpression might benefit from EGFR-targeted therapy [15–17], which motivated us greatly to initiate the deeper exploration. Previously, we have established many patient-derived xenografts (PDX) of advanced ESCC using gastroscopic biopsies [18], which faithfully resembled the original patients’ tumors and had been regarded as the optimal preclinical mouse avatar [19]. Here in this study, we utilized ESCC PDXs and cell lines to systematically identify the most promising EGFR blocker and provide a more accurate evidence for clinical trials, followed by its further investigations of underlying mechanisms, predictive biomarkers, and acquired resistant mechanisms together with reversing strategies.

## Methods

### Drugs and antibodies

Afatinib dimaleate was provided by Boehringer Ingelheim International GmbH (Germany). Gefitinib (#S1025), osimertinib (#S7297), dasatinib hydrochloride (#HY-10181A), and crizotinib hydrochloride (#HY-50878A) were purchased from Selleck Chemicals or MedChem Express. Cetuximab (#205923-56-4) and nimotuzumab (#828933-51-3) were separately obtained from Merck (Germany) and Biotech Pharma Co., Ltd. (China). Afatinib was dissolved in water, and the other drugs were dissolved in dimethyl sulfoxide (DMSO, #0231, Amresco) for in vitro studies. Antibodies are listed in Additional file 1: Materials and Methods.

### Cell lines and cell culture

Human ESCC cell lines EC109, KYSE450, KYSE140, KYSE510, TE-1, and TE-10 were purchased from the cell bank of Peking Union Medical College (Beijing, China). Cells were cultured in RPMI 1640 medium (Gibco) supplemented with 10% FBS (Gibco) in a humidified incubator with 5% CO_2_ at 37 °C. All cell lines were confirmed by short-tandem repeat (STR) analysis and no mycoplasma contamination certified by using a Mycoplasma Detection Kit (#FM311–01, TransGen Biotech, China).

### Cell proliferation assay

A total of 3000–5000 cells per well were seeded in 96-well plates and treated with drugs. Seventy-two hours after treatment, CCK-8 (#CK04, Dojindo, Japan) was added to assess cell viability, and the absorbance at 450 nm was measured on a Microplate Absorbance Reader (Bio-Rad). The IC50 was calculated using GraphPad software. All assays were repeated at least three times.

### Generation of afatinib-resistant cell lines

EC109 and KYSE450 cells were cultured with stepwise escalating concentrations of afatinib, starting at 100 nM and increasing to 2 μM (EC109) or 5 μM (KYSE450), at which cells could proliferate. Control cells were parallel treated with vehicle. Cell proliferation assays were conducted to confirm the resistance, and parental analysis was performed by STR genotyping.

### Cell cycle and apoptosis analysis

Cells were treated with afatinib or vehicle for 48 h. Apoptosis was measured by PE Annexin V Apoptosis Detection Kit I (#559763, BD Pharmingen), and cell cycle distribution was performed using PI/RNase staining buffer solution (#550825, BD Pharmingen) according to manufacturer’s instructions. The results were analyzed using FlowJo 7.6 software and ModFit LT 4.0 software.

### Animal experiments in mouse

All animal studies were approved by the Ethics Committee of Animal Experiments of Peking University Cancer Hospital and were conducted in compliance with the Guide for the Care and Use of Laboratory Animals of the National Institutes of Health. For cell line-derived xenografts (CDX), five million cells were injected subcutaneously into one flank of 6-week-old non-obese diabetic/severe combined immunodeficiency (NOD/SCID) female mice (Beijing HFK Bioscience Co., Ltd., China). The PDX models were established and passed serially as described previously [18, 20, 21]. When xenografts reached 200–300 mm^3^, mice were randomly assigned to different groups with five mice per group. Dissolution and administration methods of all drugs were described in Additional file 1: Materials and Methods. Tumor size and body weight were measured every 3 days. Tumor volume was determined using the formula volume = (length × width^2^)/2, where length and width were the long and short diameters of the tumor, respectively. Tumor growth inhibition (TGI) rate was determined using the formula TGI = (1–ΔT/ΔC) × 100%, where ΔT is the change of tumor volume in the treatment group on the final day of the study and ΔC is the change of tumor volume in the control group.

### Generating the afatinib-resistant PDX model

Afatinib-sensitive PDX03 was chosen to establish the afatinib-refractory PDX model. When tumor volumes reached 200–300 mm^3^, the mice were given 15 mg/kg/day of afatinib until the fast growth of tumor after afatinib exposure for ~ 8 months containing three passages, indicating that the tumor was resistant to afatinib. Resistant PDX was named PDX03-R, and the parental PDX03-P was also generated after continuous vehicle treatment.

### Western blotting

The proteins of cells or xenograft tissues were extracted and western blotting was performed as previously described [22]. The proteins were then detected by chemiluminescence using Immobilon Western Chemiluminescent HRP Substrate (#WBKLS0500, Millipore) and visualized with a chemiluminescent detection system (GE Healthcare). Protein bands were quantified by ImageJ software. All western blotting results shown are representative of at least three experiments with independent cell lysates.

### Immunohistochemistry (IHC) and hematoxylin and eosin (H&E) staining

After the mice were sacrificed, tumors were dissected and formalin-fixed paraffin embedded (FFPE) tissue blocks were generated. IHC and H&E staining were performed as described previously [23] and were interpreted by two pathologists in our hospital independently. Ki-67 scoring was in accordance with a previous report [24]. Scoring for EGFR, pERK, and pS6 used the following scale: 0 = no staining, 1+ = weak or focal staining, 2+ = moderate staining, and 3+ = strong staining.

### TaqMan copy number assays

Genomic DNA was extracted from cell lines or xenograft tissues using an EasyPure Genomic DNA Kit (#EE101, TransGen Biotech) following the manufacturer’s instructions. DNA was then subjected to *EGFR* copy number analysis using TaqMan Copy Number Assays (*EGFR* Hs02925916_cn, #4400291, ThermoFisher) on an ABI 7500 FAST real-time PCR system (Applied Biosystems). *RNase P* (#4403326, ThermoFisher) was used as the control gene. Copy number was then calculated by Copy Caller v 2.0 software using the comparative Ct (ΔΔCt) method. Normal human control DNA (#4312660, ThermoFisher) was used as the reference. When the relative copy number was ≥ 3.0, the *EGFR* copy number was determined to be gained.

### *MET* knockdown

The *MET* short-hairpin RNA (shRNA) virus was purchased from Genechem, China. Cells were seeded in a six-well plate and infected at a density of 3 × 10^5^ per well with 10 μL virus following the manufacturer’s instructions. The shRNA sequences are as follows: sh-Ctrl: TTCTCCGAACGTGTCACGT; sh-*MET*#1: TGGCTGGTGGCACTTTACTTA; sh-*MET*-#2: GAGGGACAAGGCTGACCATAT.

### Next-generation panel sequencing

Next-generation panel sequencing and subsequent data analysis were performed as by Novogene, Beijing and were described in Additional file 1: Materials and Methods.

### Transcriptome sequencing (RNA-seq)

Total mRNA was extracted using TRIzol reagent (#15596018, Invitrogen) according to the manufacturer’s instructions. RNA-seq and subsequent data analysis were performed by Novogene Bioinformatics Institute (China) and were described in Additional file 1: Materials and Methods.

### Statistical analysis

Statistical analyses were performed using SPSS 23.0 software or Graphpad software. Differences were analyzed using unpaired two-tailed *t* tests (two groups) or one-way ANOVA analysis (more than two groups). All data are presented as means ± SDs. *P* values < 0.05 were considered significant for all analyses.

## Results

### Afatinib demonstrates greater anti-tumor activity than other generation EGFR-TKIs or mAbs in ESCC in vitro and in vivo

We first evaluated six ESCC cell lines and two established ESCC PDXs for their in vitro and in vivo sensitivity to the first-, second-, and third-generation EGFR-TKIs (namely gefitinib, afatinib, and osimertinib, respectively) and two EGFR-mAbs (cetuximab and nimotuzumab), respectively. As shown in Additional file 2: Table S1 and Additional file 3: Figure S1A, for in vitro cells, EGFR-mAbs exhibited limited anti-proliferative activities, and EC109, KYSE450, and KYSE140 cells were more sensitive to EGFR-TKIs than the other three cells. Meanwhile, the second-generation EGFR-TKI, afatinib, exerted stronger anti-proliferative activities than gefitinib or osimertinib in EC109, KYSE450, and KYSE140 cells. For in vivo PDXs, afatinib also exhibited the greatest anti-tumor effects among the EGFR blockers, with a TGI of 100.22% for PDX03 and 82.65% for PDX06 (Additional file 3: Figure S1B).

**Fig. 1:**
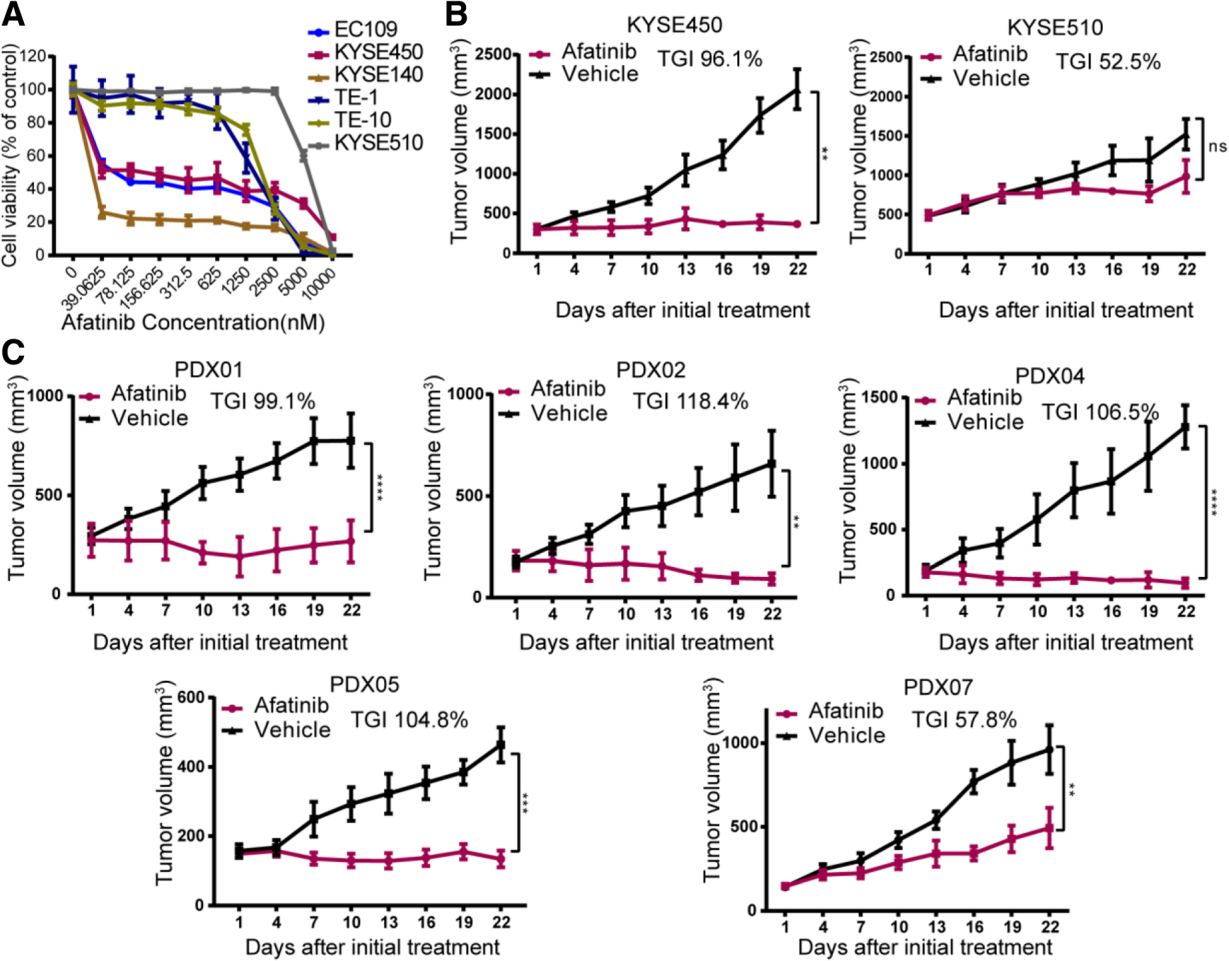
Afatinib demonstrates greater anti-tumor activity than other generation EGFR-TKIs or mAbs in ESCC. **a** Six ESCC cell lines were treated with afatinib at the indicated concentrations (from 0 to 10 μM) for 72 h. Cell viability relative to vehicle-treated controls is shown (means ± SDs; three independent assays). **b** Tumor growth curves show the in vivo assessment of afatinib-sensitive KYSE450 and afatinib-insensitive KYSE510 cells treated with vehicle control or afatinib (15 mg/kg/day, oral gavage, *n* = 5) for 21 days. **c** The efficacy of afatinib was further explored in another five PDXs for 21 days treatment (15 mg/kg/day, oral gavage, *n* = 5). Tumor growth curves and corresponding TGI are shown here. Data are presented as means ± SDs. *P* values were calculated using one-way ANOVA or unpaired two-tailed *t* tests. **P < 0.01; ***P < 0.001; ****P < 0.0001; ns = not significant

Based on the above results, the inhibitory effects of very promising afatinib were further validated in two cell-derived xenografts and five another ESCC PDXs. Figure 1a showed that EC109, KYSE450, and KYSE140 cells were more sensitive to afatinib than KYSE510, TE-1, and TE-10 cells in vitro. Therefore, we selected KYSE450 and KYSE510 cells to evaluate the sensitivity to afatinib in vivo. Consistent with the in vitro assay, afatinib significantly inhibited the growth of KYSE450 xenografts (TGI, 96.1%) but had a limited inhibitory effect on KYSE510 xenografts (TGI, 52.5%; Fig. 1b). Moreover, four out of five PDXs showed cessation or shrinkage of tumor growth with TGIs ranging from 99.1 to 118.4% thereby illustrating a high sensitivity to afatinib (Fig. 1c). However, PDX07 showed tumor increase under afatinib treatment with a TGI of 57.8%, which illustrated a low sensitivity to afatinib.

### *EGFR* CNG or overexpression predicts a higher sensitivity to afatinib

**Fig. 2:**
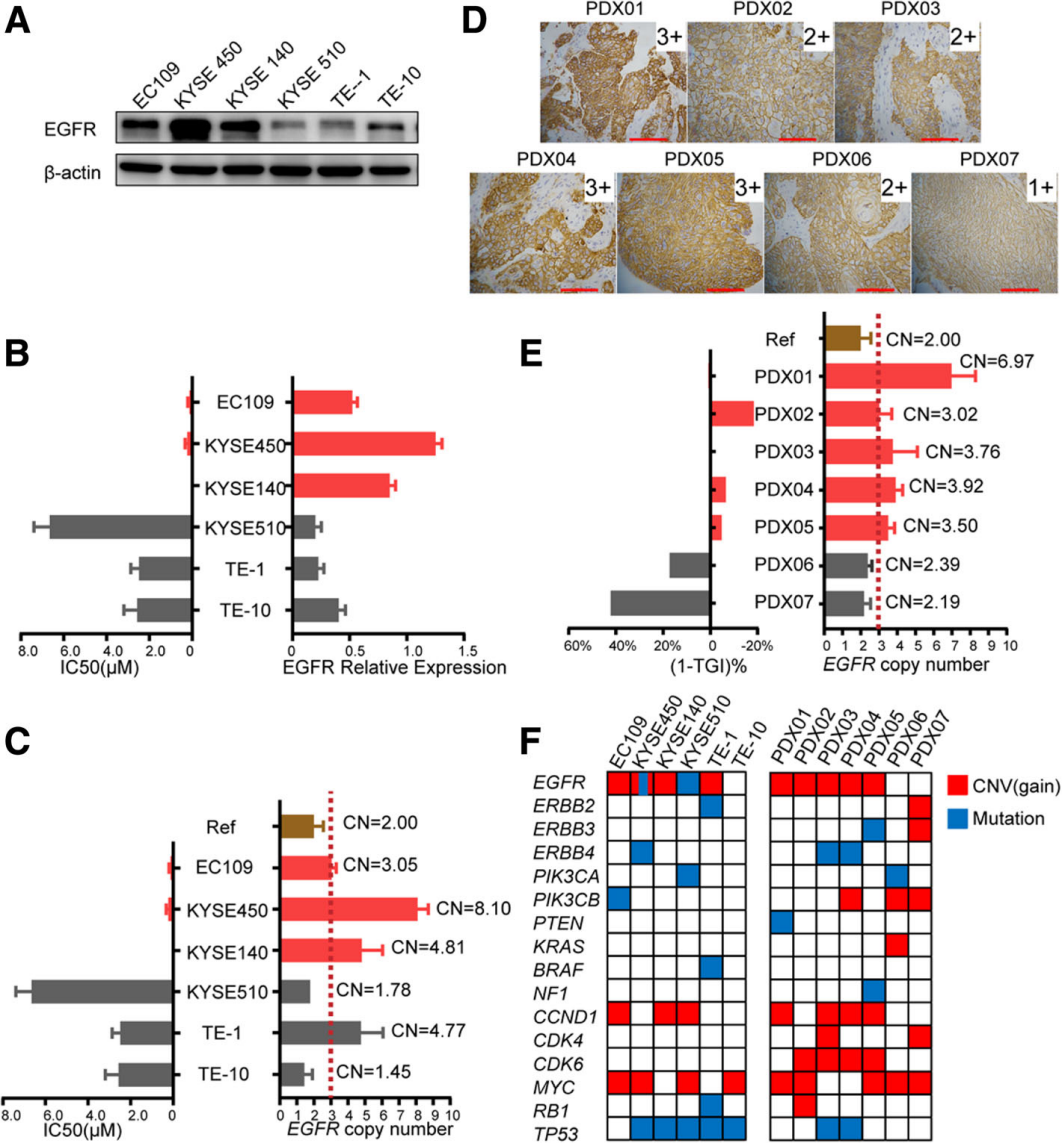
*EGFR* CNG or overexpression predicts a higher sensitivity to afatinib. **a** Western blotting showed the basal protein levels of EGFR expression in six ESCC cell lines. **b** The relative EGFR expression across six ESCC cell lines was calculated according to the above western blotting after normalized to β-actin using ImageJ software. The bar chart of IC50 was drawn using the data in Additional file 2: Table S1. EC109, KYSE450, and KYSE140 are drawn in red while KYSE510, TE-1, and TE-10 are drawn in gray according to their sensitivity to afatinib. **c** The *EGFR* copy number of the six ESCC cell lines was detected using copy number assays. A copy number ≥ 3 was defined as an *EGFR* copy number gain. Data are presented as means ± SDs of three independent assays. CN, copy number; Ref, normal human control DNA . **d** The expression of EGFR in seven ESCC PDXs was detected by IHC (× 200 magnification; scale bars = 100 μM). **e** This bar chart demonstrated ESCC PDXs with *EGFR* CNG were more sensitive to afatinib treatment.1-TGI% was calculated using the data in Additional file 3: Figure S1B and Fig. 1c. When 1-TGI% was close to 0 or a negative value, the xenografts showed growth cessation or shrinkage. When 1-TGI% was a positive value or far greater than 0, the xenografts exhibited tumor growth. Data are presented as means ± SDs of three independent assays. **f** The main genetic features of ESCC cell lines and PDX models were detected using next-generation panel sequencing. Only genes in EGFR-related pathways or important tumor suppressor genes are listed. Mutations containing single nucleotide variant (SNV) and InDel are depicted in blue whereas CNV (only copy number gain) is depicted in red

Since different ESCC cell lines and PDXs demonstrated a wide range of sensitivity to afatinib, the potential predictive biomarkers for EGFR-targeted therapy were investigated. Western blotting showed EGFR was the major molecule among the four pan-HER family members which expressed in the six ESCC cell lines (Fig. 2a), whereas HER2, HER3, and HER4 were hardly detected. The expression level of EGFR in EC109, KYSE450, and KYSE140 cells, which were sensitive to afatinib, was higher than that in other cells (Fig. 2). Meanwhile, these three afatinib-sensitive cell lines had an *EGFR* copy number gain, with copy numbers of 3.05, 8.10, and 4.81, respectively (Fig. 2c). A significant reverse correlation was found between EGFR expression or copy number and afatinib sensitivity as indicated by IC50 (Fig. 2b and c), which was further validated in PDXs. PDXs (PDX01–05) with cessation or shrinkage of tumor growth after afatinib treatment showed a high EGFR expression (IHC score of 3+ or 2+, Fig. 2d) and a copy number > 3 (Fig. 2e). Also, a very good correlation was presented between *EGFR* copy number and inhibitory effects of afatinib in PDXs (Fig. 2e). We further explored whether other genomic alterations could affect the response of these models to afatinib. Panel sequencing of these cell lines and PDXs showed that KYSE450 harbored an activating *EGFR* mutation (S7681), TE-1 harbored an activating *BRAF* mutation at codon 326 (I326V), and KYSE510 and PDX06 harbored an activating *PIK3CA* mutation (E545K and H1047L, respectively), which partially provided rationale for the sensitivity and resistance of these models to afatinib (Fig. 2f). Importantly, there was high consistency in *EGFR* CNV between next-generation sequencing and copy number assays. Together, these results provided a convictive evidence for using *EGFR* CNG or overexpression as a predictive biomarker in future clinical trial design.

### Afatinib plays inhibitory effects by blocking EGFR phosphorylation and downstream signaling pathways as well as inducing cell cycle arrest and apoptosis

**Fig. 3:**
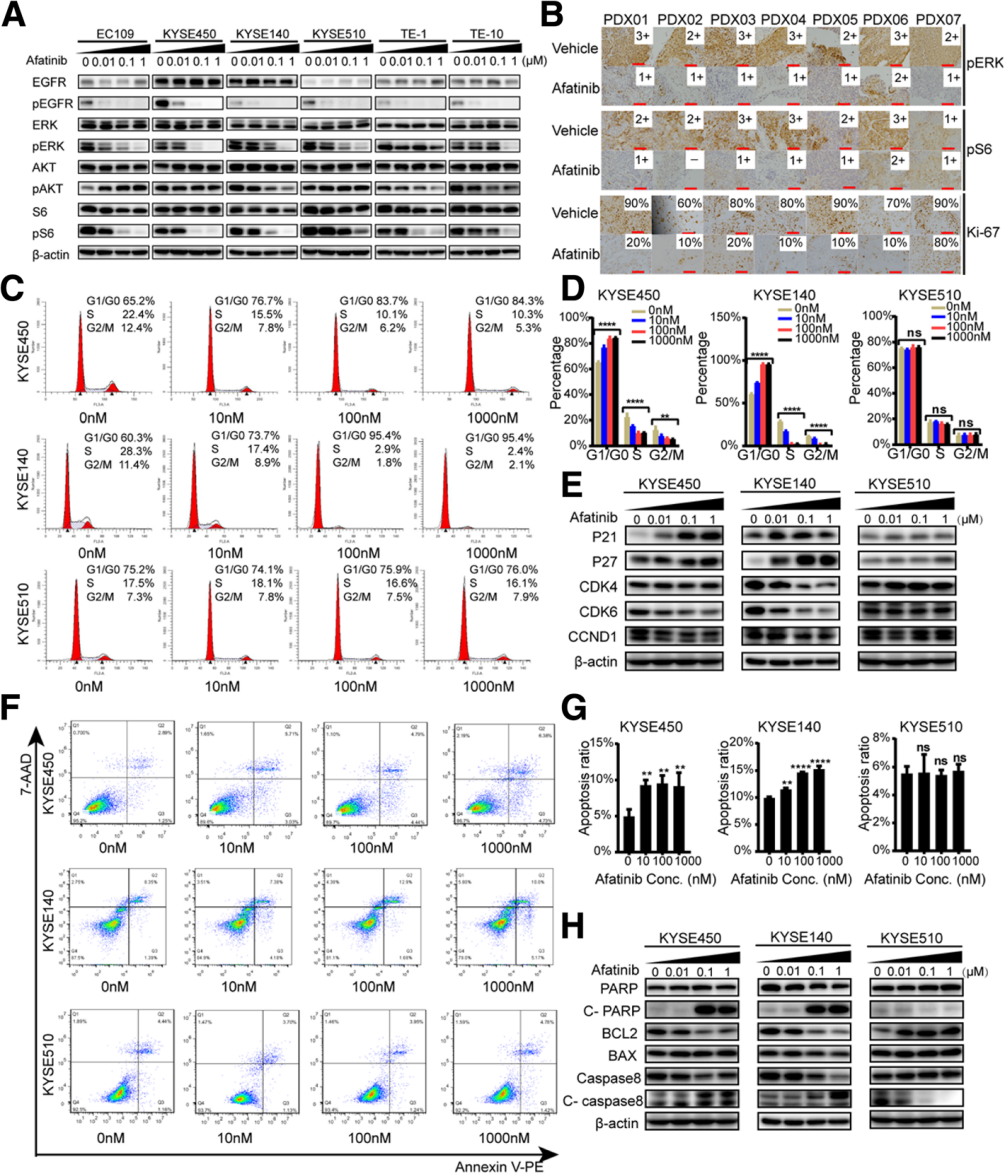
The anti-tumor mechanisms of afatinib in ESCC cell lines and PDXs. **a** Six ESCC cell lines were treated with 0, 10 nM, 100 nM, and 1 μM afatinib and harvested after 48 h. Immunoblots show the response of EGFR downstream signaling molecules to afatinib. **b** IHC staining for pERK, pS6, and Ki-67 in seven PDXs tumors after 21 days treatment. Representative images and interpretation (by two independent pathologists) are shown (× 200 magnification; scale bars = 100 μM). **c–h** KYSE450, KYSE140, and KYSE510 cells were treated with 0, 10 nM, 100 nM, and 1 μM afatinib after serum-starvation for 12 h. After 48 h of treatment, the cells were harvested and assayed as described below. The effects of afatinib on cell cycle distribution were assessed using flow cytometry after PI/RNase staining (**c**). The distribution of cells in the cell cycle is depicted (**d**). G1 phase-associated proteins (P21, P27, CDK4, CDK6, and CCND1) were assessed using western blotting (**e**). Flow cytometry showed the apoptosis induced by afatinib treatment using PE-annexin V and 7-AAD staining (**f**). The percentage of cells in early apoptosis (Q3) and late apoptosis (Q2) was calculated as the total apoptosis ratio (**g**). Apoptosis-related proteins (c-PARP, c-caspase8, BCL2, and BAX) were measured by western blotting after afatinib treatment (**h**). Data are presented as means ± SDs of three independent assays. *P* values were calculated using one-way ANOVA or unpaired two-tailed *t* tests.*P < 0.05; **P < 0.01; ***P < 0.001; ****P < 0.0001; ns = not significant

We next evaluated the biochemical effects of afatinib in these ESCC cell lines and PDXs. EGFR phosphorylation was effectively blocked by 10 nM afatinib in all cell lines (Fig. 3a). S6 and ERK phosphorylation (pS6 and pERK) were significantly inhibited by 100 nM afatinib in the afatinib-sensitive lines EC109, KYSE450, and KYSE140, but 100 nM afatinib did not inhibit pS6 in KYSE510 or pERK in TE-1 cells possibly due to the abovementioned PIK3CA and BRAF mutations, respectively (Fig. 3a). Phosphorylated AKT (S473) was either inhibited or activated among different cell lines (Fig. 3a), which was possibly due to the feedback bypass activation [25] and need to be further investigated. Similarly, afatinib effectively inhibited pS6 and pERK in PDXs tissue as detected by IHC (Fig. 3b). Moreover, afatinib significantly decreased the expression level of Ki-67 and further verified its anti-proliferative activities (Fig. 3b). In addition, afatinib could induce obvious G1 phase arrest and apoptosis in a dose-dependent manner in afatinib-sensitive KYSE450, KYSE140, and EC109 cells, but not in afatinib-insensitive KYSE510 cells (Fig. 3c, d, f, and g; Additional file 4: Figure S2A, S2B, S2D, and S2E). Consistent with G1 phase arrest evaluated by flow cytometry, levels of P21 and P27, two negative regulators of the cell cycle, were increased, whereas CDK4, CDK6, and CCND1 were decreased in KYSE450, KYSE140, and EC109 cells other than KYSE510 cells after afatinib treatment (Fig. 3e and Additional file 4: Figure S2C). Also, along with cell apoptosis, cleaved form of caspase-8 and PARP were increased, and the anti-apoptotic protein BCL2 was decreased in KYSE450, KYSE140, and EC109 cells other than KYSE510 cells under afatinib treatment (Fig. 3h and Additional file 4: Figure S2F).

### Distinct differences are presented in afatinib-refractory ESCC models compared with its parental sensitive models

**Fig. 4:**
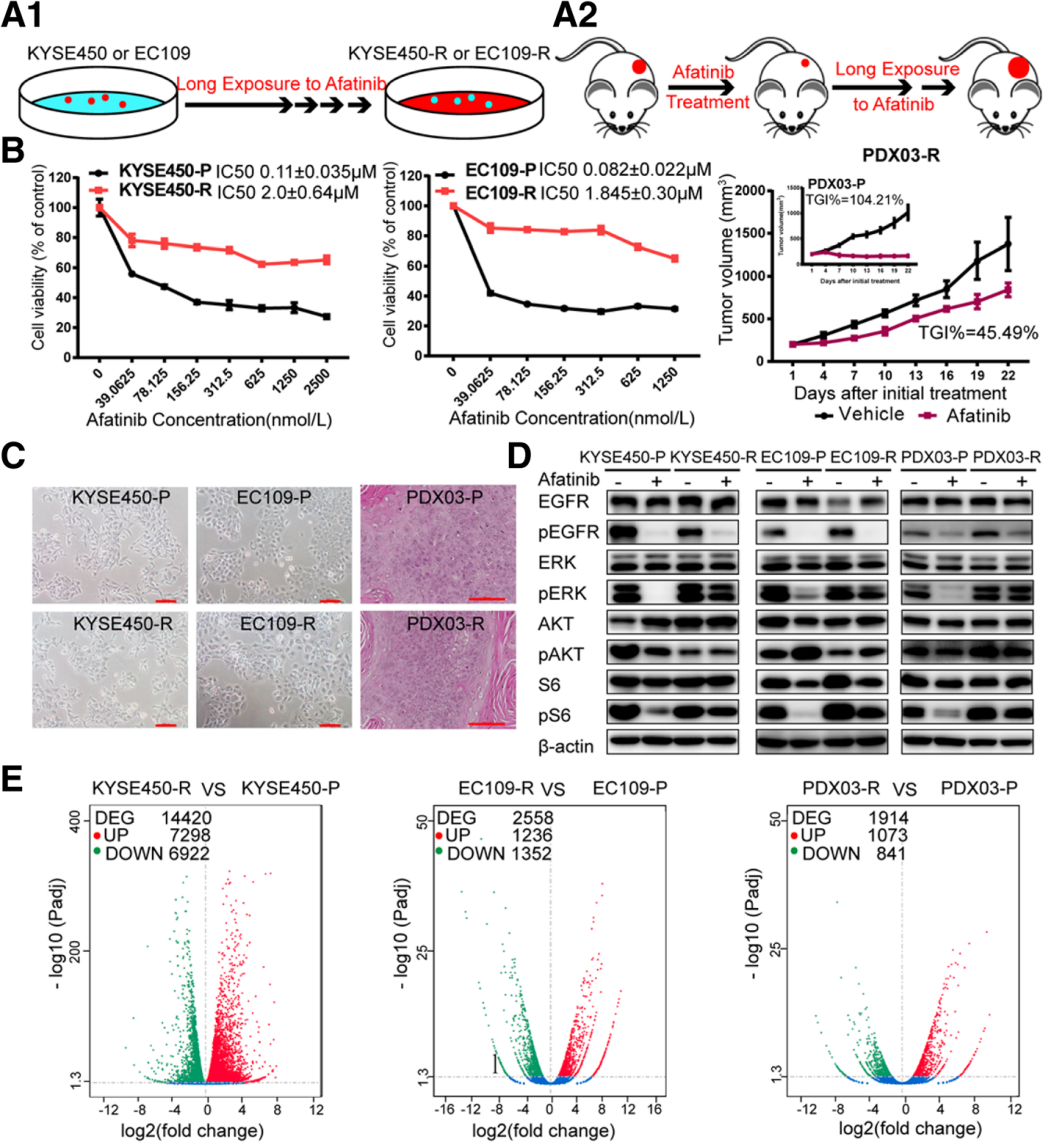
Distinct differences are presented in afatinib-refractory ESCC models compared with its parental sensitive models. **a** The cartoon depicts the process of generating two afatinib-resistant ESCC cell lines in vitro (**a1**) and one afatinib-resistant ESCC PDX in vivo (**a2**). **b** Dose-response curves were generated to confirm the resistant phenotype of KYSE450-R and EC109-R after 72 h of afatinib treatment. Data are presented as means ± SDs of three independent assays. In vivo xenograft experiments were conducted to confirm the resistant phenotype of PDX03-R. PDX03-P and PDX03-R were treated with afatinib for 21 days by oral gavage at a dose of 15 mg/kg/day (*n* = 5). Data are presented as means ± SDs. **c** Representative images of resistant and parental cells or PDX (× 100 magnification for cells; × 200 magnification for PDX; scale bars = 100 μM). The morphology of the resistant PDX model was detected by H&E staining. **d** Responses of the EGFR downstream signaling to afatinib treatment in parental and resistant cells or PDX. Cells were harvested after 200 nM afatinib treatment for 48 h. Tissue lysates were extracted from PDX03-R and PDX03-P xenografts after 21 days of afatinib treatment. All experiments were repeated three times independently. **e** Volcano plots showed the distinct differential gene profiles after the acquisition of a resistant phenotype, as detected by RNA-seq. Red dots represented upregulated genes and green dots indicated downregulated genes in resistant models compared with their counterparts. DEG, differential genes; UP, upregulated genes; DOWN, downregulated genes

Undergoing a long-term exposure to afatinib (Fig. 4a), two afatinib acquired resistant cell lines and one afatinib-acquired resistant PDX model were established and named as KYSE450-R, EC109-R, and PDX03-R, respectively. Compared with their parental cells or PDX named as KYSE450-P, EC109-P, and PDX03-P, refractory models demonstrated obvious resistance to afatinib (Fig. 4b) indicated as significantly increased IC50 (about 20 folds) in cells and decreased TGI (decreased by 58.72%) in PDX. Besides, no obvious morphological changes were obtained between afatinib-acquired resistant models and their parental sensitive models (Fig. 4c). In afatinib-refractory cells or PDX, phosphorylated S6 and ERK could no longer be inhibited by afatinib in contrast with their sensitive counterparts (Fig. 4d), which also verified the success of afatinib-acquired resistant models. Transcriptome sequencing results indicated that compared with their parental sensitive cells or PDX, afatinib-resistant models showed distinct differential gene profiles (Fig. 4e) which involved in many signaling pathways (Additional file 5: Figure S3A-C). In addition, variant analysis did not reveal any new mutations within the EGFR kinase domain or mutations in the downstream effectors like *PIK3CA*, *KRAS*, or *BRAF* after afatinib-acquired resistance (data not shown). However, the transcriptome sequencing data failed to help us find a specific gene or pathway to rationally explain the mechanisms of acquired resistance here.

### The common emergence of MET upregulation does not confer afatinib-acquired resistance in ESCC

**Fig. 5:**
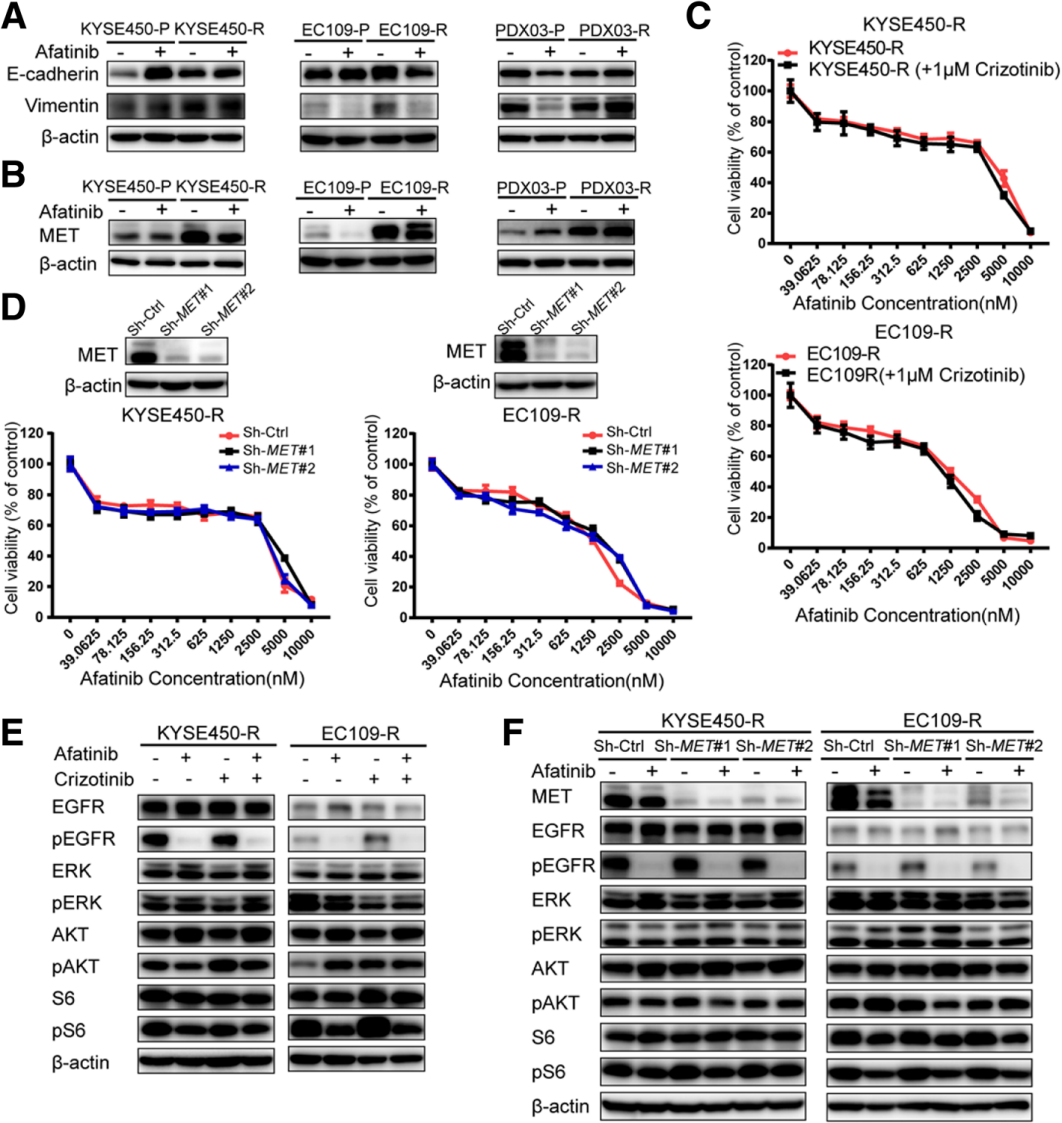
The common emergence of MET upregulation does not confer afatinib-acquired resistance in ESCC. **a** The levels of the EMT markers (E-cadherin and vimentin) between resistant and parental models. **b** MET expression was upregulated in all three afatinib-resistant models. Cells were harvested after treatment with 200 nM afatinib for 48 h. The PDX lysates used were the same as those described in Fig. 4d. All assays were repeated three times independently. **c** KYSE450-R and EC109-R cells were treated with increasing concentrations of afatinib in the presence or absence of 1 μM crizotinib for 72 h, and CCK-8 assays were performed to assess cell viability. Data are presented as the means ± SDs of three independent assays. **d** After *MET* was knocked down in KYSE450-R and EC109-R, cells were treated with increasing concentrations of afatinib for 72 h, and then CCK-8 assays were performed to assess cell viability. Data are presented as the means ± SDs of three independent assays. **e** Resistant cells were treated with 200 nM afatinib alone or in combination with 1 μM crizotinib for 48 h. **f** Resistant cells after *MET* knockdown were treated with 200 nM afatinib for 48 h. All experiments were repeated three times independently

Previous studies suggested that the epithelial to mesenchymal transition (EMT) lead to acquired resistance to EGFR TKIs in lung cancer and ESCC [8, 10]. However, the EMT process was proved not to play an important role in this study (Fig. 5a). To elucidate whether a bypass signaling pathway participated in the acquisition of resistance, the expression of some RTKs was examined, and MET upregulation was observed in all three resistant models (Fig. 5b), which suggested the possible role of MET in afatinib-acquired resistance. Crizotinib, a MET inhibitor, was added to afatinib-resistant KYSE450-R and EC109-R cells, but no any difference was found compared with afatinib monotherapy (Fig. 5c). An alternative strategy was employed by *MET* knockdown, and results also confirmed that MET downregulation could not re-sensitize the resistant cells to afatinib (Fig. 5d). Deep-going analysis demonstrated that neither combining crizotinib with afatinib nor *MET* knockdown had effects on phosphorylated S6 and ERK (Fig. 5e and f), which provided a solid evidence that targeting MET was not a strategy for overcoming acquired resistance to afatinib in ESCC.

### Increased phosphorylation of Src family kinases leads to acquired afatinib resistance in ESCC

**Fig. 6:**
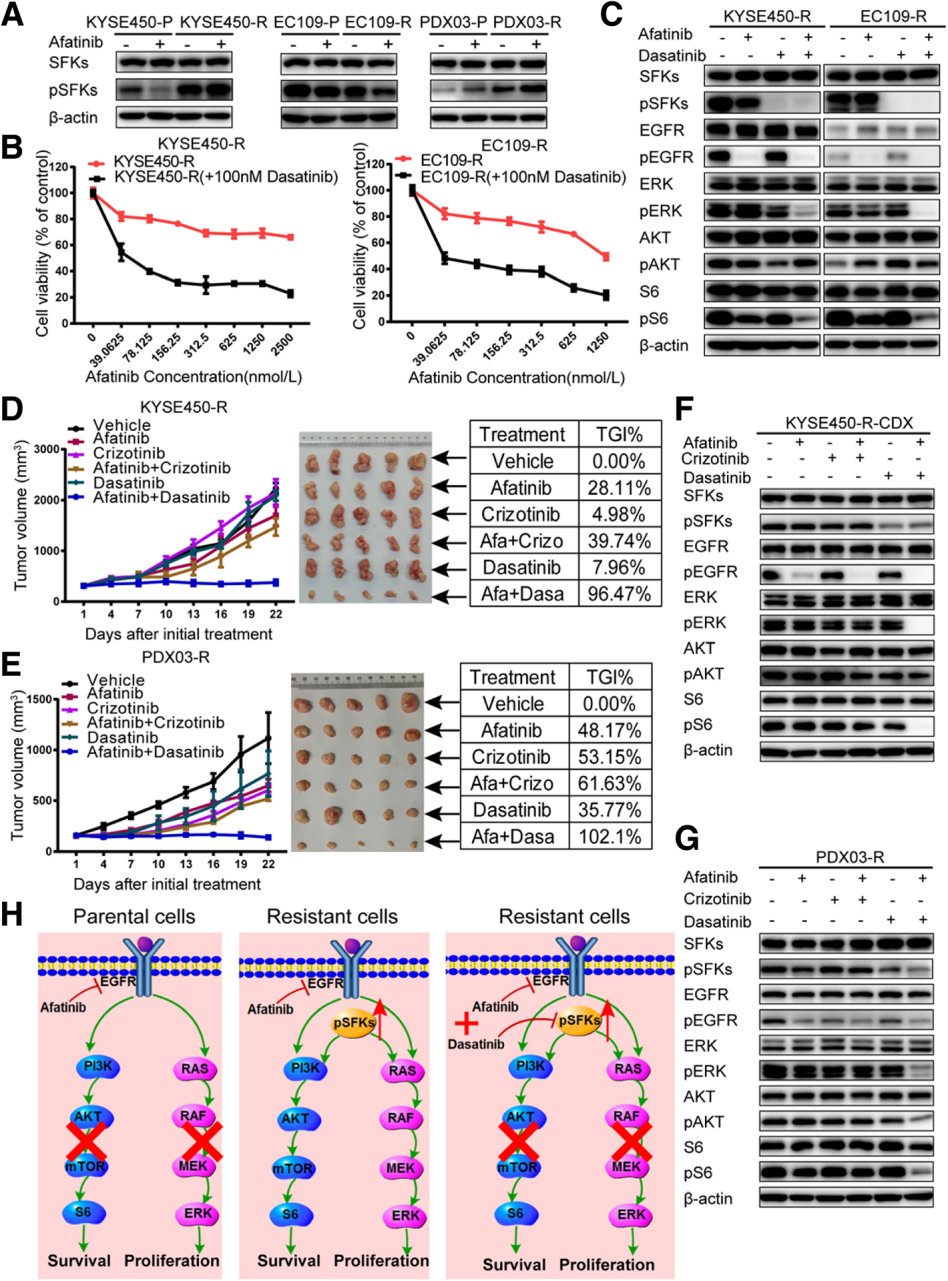
Increased phosphorylation of Src family kinases leads to acquired afatinib resistance in ESCC. **a** Increased pSFKs levels were observed in KYSE450-R and PDX03-R resistant models, but total SFKs levels were unchanged. Cells were harvested after treatment with 200 nM afatinib for 48 h. The PDX lysates used were the same as those described in Fig. 4d. All assays were repeated three times independently. **b** Resistant cells were treated with the indicated concentrations of afatinib in the presence or absence of 100 nM dasatinib for 72 h, and CCK-8 assays were performed to assess cell viability. Data are presented as the means ± SDs of three independent assays. **c** KYSE450-R and EC109-R cells were treated with 200 nM afatinib alone or in combination with 100 nM dasatinib for 48 h. **d**, **e** Curves showing the xenografts growth of KYSE450-R (**d**) and PDX03-R (**e**) treated with vehicle control, afatinib (15 mg/kg), crizotinib (25 mg/kg), afatinib (15 mg/kg) plus crizotinib (25 mg/kg), dasatinib (15 mg/kg), or afatinib (15 mg/kg) plus dasatinib (15 mg/kg). Data are presented as means ± SDs; *n* = 5. Mice were sacrificed after 21 days of treatment, and xenografts were isolated. Pictures of the xenografts are shown with the corresponding TGI listed in the tables. **f**, **g** Lysates were extracted from KYSE450-R (**f**) and PDX03-R (**g**) xenografts after 21 days of treatment with the corresponding inhibitors and analyzed by western blotting to explore the downstream signaling responses. The lysates were then probed with the indicated antibodies. All experiments were repeated three times independently. **h** A schematic of the molecular mechanisms of acquired resistance revealed in this study

We then turned to Src family kinases (SFKs), as SFKs are well-known upstream regulators of PI3K and MAPK pathways [26]. Western blotting showed increased SFKs phosphorylation at Tyr-416 in the KYSE450-R and PDX03-R models but not in EC109-R (Fig. 6a). However, no obvious upregulation in total SFKs levels was observed. Therefore, we explored whether increased pSFKs levels lead to the acquired resistance. The SFKs inhibitor, dasatinib, could re-sensitize KYSE450-R and EC109-R cells to afatinib treatment (Fig. 6b), which was validated by results that dasatinib combined with afatinib could significantly inhibit phosphorylated S6 and ERK (Fig. 6c), although no obvious increase of pSFKs was observed in EC109-R. These data were further confirmed in vivo xenografts. As shown in Fig. 6d and e, compared with any single drug or afatinib combined with crizotinib, a combination of afatinib with dasatinib could greatly inhibit the growth of xenografts derived from KYSE450-R and PDX03-R, with TGIs of 96.47% and 102.10%, respectively. Further analysis also verified the in vivo results indicated as the inactivation of phosphorylated S6 and ERK after afatinib combined with dasatinib treatment (Fig. 6f and g). Based on our results, the molecular mechanisms that afatinib works or not followed by the subsequential strategies are depicted schematically in Fig. 6h.

## Discussion

The present study demonstrated that afatinib exerted greater anti-tumor effects on ESCC cell lines and PDXs than other-generation EGFR-TKIs or mAbs in vitro and in vivo. Previous in vitro kinase assays revealed that afatinib had a higher affinity for wild-type EGFR than gefitinib or osimertinib [27, 28], which were consistent with the current findings that afatinib exhibited better efficacy than other EGFR blockers in ESCC because of its higher potency for wild-type EGFR and broader irreversible ErbB blockade compared with inhibitors that block EGFR alone. Two clinical trials uncovered that afatinib achieved better clinical improvements than erlotinib in lung squamous cell carcinoma (LUSC) [29] or methotrexate in head and neck squamous cell carcinoma (HNSCC) [30], illustrating afatinib’s outstanding clinical efficacy in tumors with wild-type EGFR. Given the similar genomic landscape among ESCC, HNSCC, and LUSC [5], we speculate that afatinib will have promising performance in select ESCC patients. The current study provided encouraging evidence for evaluating afatinib in the clinical trials for ESCC patients.

Previous clinical trials of gefitinib (the COG study) [14, 15] and icotinib (first-generation EGFR-TKIs) [31] demonstrated that EGFR-TKIs exhibited favorable efficacy in ESCC patients with *EGFR* amplification as detected by fluorescence in situ hybridization (FISH) method. Despite with different detection methods for *EGFR* copy number, we showed that *EGFR* CNG, as detected by copy number assays, was proved to be the predictor for afatinib efficacy, which further verified the role of EGFR as a potential target in ESCC and was consistent with what we have found in gastric cancer [21]. However, TE-1 cells with *EGFR* CNG were not sensitive to afatinib. Further genomic analysis showed that in addition to *EGFR* CNG, TE-1 harbored a *BRAF* mutation at codon 326 (I326V), which had been reported to result in primary resistance to panitumumab [32]. Besides, KYSE510 harbored a non-activating *EGFR* mutation (A702D) in the juxtamembrane region and showed no correlations with sensitivity to EGFR-TKIs according to previous studies [33]; KYSE450 harbored an activating *EGFR* mutation (S768I) in tyrosine kinase domain, and this mutation was reported to be sensitive to afatinib [34]. Since afatinib is a pan-HER inhibitor, we also examined the expression levels and genetic alterations of HER2, HER3, and HER4 in these ESCC cell lines and PDX models, but all these three molecules were hardly detected by western blotting or IHC, and no genetic alterations were found to be correlated with the afatinib sensitivity (data not shown). Above all, *EGFR* CNG or amplification may be a promising predictive biomarker for EGFR-targeted therapy in ESCC patients, but patients with mutations in EGFR downstream effectors such as *PIK3CA* or *BRAF* may exhibit de novo resistance to afatinib.

Despite the initial response of most targeted agents, resistance inevitably emerges. Previous studies revealed several mechanisms of acquired resistance to EGFR inhibitors in lung cancer such as *EGFR* T790 M mutation, *MET* amplification, IGF-1R upregulation, AXL upregulation, or histologic changes like transformation to small-cell lung cancer or EMT [35].However, the mechanisms of acquired resistance to EGFR-targeted therapy in an ESCC setting, where *EGFR* is wild-type, are poorly understood. Zhou et al. [8] showed that EMT mediated the acquired resistance to erlotinib in ESCC cell lines. However, EMT did not play a key role in the acquisition of resistance in our resistant models. After excluding the emergence of mutations in *EGFR *and downstream effectors such as *PIK3CA*, *KRAS*, and *BRAF*, we speculated that bypass signaling pathways might result in the resistance. We first focused on MET for its upregulation in all three resistant models, but it was finally proved to be irrelevant to the resistance in vitro and in vivo. Others [36] also found that MET upregulation without amplification was not associated with acquired resistance to EGFR-TKIs in lung cancer, but instead only enhanced migratory and invasive abilities, which was consistent with the current findings.

SFKs are a group of non-receptor tyrosine kinases containing nine members and are well-known upstream regulators of PI3K and MAPK pathways, which play an important role in cell proliferation, survival, adhesion, and invasion during tumor development [37]. Takeshi et al. [38] and Eiki et al. [25] revealed that SFKs activation could mediate resistance to EGFR-TKIs and suggested that concomitant inhibition of SFKs and EGFR could overcome this resistance in lung cancer. Here, we showed that SFKs phosphorylation at Tyr-416 was increased in the afatinib-refractory ESCC models without upregulation of total SFKs levels. Dual EGFR and SFKs blockade could abolish the downstream phosphorylation of S6 and ERK and therefore overcome the resistance. In Eiki’s study, they found that increased pSFKs levels were caused by amplification of *YES1*, one member of the SFKs, and further led to EGFR-TKIs resistance [25]. However, since the total protein levels of SFKs or the transcriptional levels of the nine SFKs members as indicated by RNA-seq (data not shown) were unchanged, we did not further explore which member of the nine SFKs lead to such resistance. Future studies need to elucidate the reasons for the increased pSFKs levels in our resistant models, and explore whether dual EGFR/SFKs blockade could delay the emergence of resistance.

## Conclusions

In conclusion, our work is the first attempt to compare the efficacy of different EGFR blockers using ESCC preclinical models in vitro and in vivo. We found that afatinib was a better choice for ESCC, and *EGFR* CNG or overexpression was recommended as a predictive biomarker for EGFR-targeted therapy in ESCC patients. Afatinib can inhibit EGFR downstream pathways as well as inducing apoptosis and G1 phase arrest in ESCC preclinical models. In addition, activated SFKs could mediate acquired resistance to afatinib and dual EGFR/SFKs blockade can overcome this resistance in an ESCC setting, which need to be further validated in clinical practice.

## Additional files

Additional file 1: Materials and Methods. (DOCX 22 kb)

Additional file 2: **Table S1.** Efficacy of different EGFR blockers on ESCC cell lines. (DOCX 17 kb)

Additional file 3: **Figure S1.** Efficacy of different EGFR blockers on ESCC cell lines and PDX models. (A) Six ESCC cell lines were treated with different EGFR blockers (gefitinib, afatinib, osimertinib, cetuximab, and nimotuzumab) at the indicated concentrations (from 0 to 10 μM for TKIs and 0–1000 μg/mL for mAbs). After treatment for 72 h, cell growth inhibition was detected using CCK-8 assays. Cell viability at different doses relative to vehicle-treated controls is shown (means ± SD; three independent assays). (B) Curves plots the growth of PDX03 and PDX06 treated with vehicle control, gefitinib (50 mg/kg/day, oral gavage), afatinib (15 mg/kg/day, oral gavage), osimertinib (15 mg/kg/day, oral gavage), cetuximab (0.5 mg per mouse, twice a week, i.p.), or nimotuzumab (0.5 mg per mouse, twice a week, i.p.). Mice were sacrificed after 21 days of treatment and xenografts were isolated. Pictures of the xenografts are shown and the corresponding TGI is listed in the tables. Data are presented as means ± SDs; *n* = 5. (DOCX 1320 kb)

Additional file 4: **Figure S2.** Effects of afatinib on cell cycle and apoptosis in EC109 cells. EC109 cells were treated with 0, 10 nM, 100 nM, and 1 μM afatinib after serum-starvation for 12 h. After 48 h treatment, the cells were harvested and assayed as described below. The effects of afatinib on cell cycle distribution were assessed using flow cytometry after PI/RNase staining (A). The distribution of cells in the cell cycle is depicted (B). G1 phase-associated proteins (P21, P27, CDK4, CDK6, and CCND1) were assessed using western blotting (C).Flow cytometry showed the apoptosis induced by afatinib treatment using PE-annexin V and 7-AAD staining (D). The percentage of cells in early apoptosis (Q3) and late apoptosis (Q2) was calculated as the total apoptosis ratio (E). Apoptosis-related proteins (c-PARP, c-caspase8, BCL2, and BAX) were measured by western blotting after afatinib treatment (F). Data are presented as means ± SDs of three independent assays. *P* values were calculated using one-way ANOVA or unpaired two-tailed *t*-tests.*P < 0.05; **P < 0.01; ***P < 0.001; ****P < 0.0001; ns = not significant. (DOCX 570 kb)

Additional file 5: **Figure S3.** Pathway enrichment by RNA-Seq. Dot plots showing the enrichment results of KEGG pathway analysis for KYSE450-R versus KYSE450-P (A), EC109-R versus EC109-P (B), and PDX03-R versus PDX03-P (C), as detected by RNA-seq. The size of the dots indicates the number of genes enriched in the corresponding pathways. The color of the dots indicates the significance level of the enriched pathways, as represented by the value of *P*adj. (DOCX 549 kb)

## Abbreviations

CDX: Cell line-derived xenografts
CNG: Copy number gain
CNV: Copy number variation
DMSO: Dimethyl sulfoxide
EGFR: Epidermal growth factor receptor
EMT: Epithelial to mesenchymal transition
ESCC: Esophageal squamous cell carcinoma
FISH: Fluorescence in situ hybridization
H&E: Hematoxylin and eosin
HNSCC: Head and neck squamous cell carcinoma
IHC: Immunohistochemistry
LUSC: Lung squamous cell carcinoma
mAbs: Monoclonal antibodies
NOD/SCID: Non-obese diabetic/severe combined immunodeficiency
PCR: Polymerase chain reaction
PDXs: Patient-derived xenografts
RNA-seq: Transcriptome sequencing
RTK: Receptor tyrosine kinase
SFKs: Src family kinases
shRNA: Short-hairpin RNA
STR: Short-tandem repeat
TGI: Tumor growth inhibition
TKI: Tyrosine kinase inhibitors

## References

[1] Zeng H, Zheng R, Zhang S, Zuo T, Xia C, Zou X (2016). Esophageal cancer statistics in China, 2011: estimates based on 177 cancer registries. Thorac Cancer.

[2] Rustgi AK, El-Serag HB (2014). Esophageal carcinoma. N Engl J Med.

[3] Pennathur A, Gibson MK, Jobe BA, Luketich JD (2013). Oesophageal carcinoma. Lancet.

[4] Domper Arnal MJ, Ferrandez Arenas A, Lanas Arbeloa A (2015). Esophageal cancer: risk factors, screening and endoscopic treatment in western and eastern countries. World J Gastroenterol.

[5] Song Y, Li L, Ou Y, Gao Z, Li E, Li X (2014). Identification of genomic alterations in oesophageal squamous cell cancer. Nature.

[6] Lin DC, Hao JJ, Nagata Y, Xu L, Shang L, Meng X (2014). Genomic and molecular characterization of esophageal squamous cell carcinoma. Nat Genet.

[7] Gao YB, Chen ZL, Li JG, Hu XD, Shi XJ, Sun ZM (2014). Genetic landscape of esophageal squamous cell carcinoma. Nat Genet.

[8] Zhou J, Wu Z, Wong G, Pectasides E, Nagaraja A, Stachler M (2017). CDK4/6 or MAPK blockade enhances efficacy of EGFR inhibition in oesophageal squamous cell carcinoma. Nat Commun.

[9] Wang X, Niu H, Fan Q, Lu P, Ma C, Liu W (2016). Predictive value of EGFR overexpression and gene amplification on icotinib efficacy in patients with advanced esophageal squamous cell carcinoma. Oncotarget.

[10] Rotow J, Bivona TG (2017). Understanding and targeting resistance mechanisms in NSCLC. Nat Rev Cancer.

[11] Crosby T, Hurt CN, Falk S, Gollins S, Mukherjee S, Staffurth J (2013). Chemoradiotherapy with or without cetuximab in patients with oesophageal cancer (SCOPE1): a multicentre, phase 2/3 randomised trial. Lancet Oncol.

[12] Suntharalingam M, Winter K, Ilson D, Dicker AP, Kachnic L, Konski A (2017). Effect of the addition of cetuximab to paclitaxel, cisplatin, and radiation therapy for patients with esophageal cancer: the NRG oncology RTOG 0436 phase 3 randomized clinical trial. JAMA Oncol.

[13] Lorenzen S, Schuster T, Porschen R, Al-Batran SE, Hofheinz R, Thuss-Patience P (2009). Cetuximab plus cisplatin-5-fluorouracil versus cisplatin-5-fluorouracil alone in first-line metastatic squamous cell carcinoma of the esophagus: a randomized phase II study of the Arbeitsgemeinschaft Internistische Onkologie. Ann Oncol.

[14] Dutton SJ, Ferry DR, Blazeby JM, Abbas H, Dahle-Smith A, Mansoor W (2014). Gefitinib for oesophageal cancer progressing after chemotherapy (COG): a phase 3, multicentre, double-blind, placebo-controlled randomised trial. Lancet Oncol.

[15] Petty RD, Dahle-Smith A, Stevenson DAJ, Osborne A, Massie D, Clark C (2017). Gefitinib and EGFR gene copy number aberrations in esophageal Cancer. J Clin Oncol.

[16] Janmaat ML, Gallegos-Ruiz MI, Rodriguez JA, Meijer GA, Vervenne WL, Richel DJ (2006). Predictive factors for outcome in a phase II study of gefitinib in second-line treatment of advanced esophageal cancer patients. J Clin Oncol.

[17] Ilson DH, Kelsen D, Shah M, Schwartz G, Levine DA, Boyd J (2011). A phase 2 trial of erlotinib in patients with previously treated squamous cell and adenocarcinoma of the esophagus. Cancer.

[18] Zou J, Liu Y, Wang J, Liu Z, Lu Z, Chen Z (2018). Establishment and genomic characterizations of patient-derived esophageal squamous cell carcinoma xenograft models using biopsies for treatment optimization. J Transl Med.

[19] Lai Y, Wei X, Lin S, Qin L, Cheng L, Li P (2017). Current status and perspectives of patient-derived xenograft models in cancer research. J Hematol Oncol.

[20] Zhu Y, Tian T, Li Z, Tang Z, Wang L, Wu J (2015). Establishment and characterization of patient-derived tumor xenograft using gastroscopic biopsies in gastric cancer. Sci Rep.

[21] Chen Z, Huang W, Tian T, Zang W, Wang J, Liu Z (2018). Characterization and validation of potential therapeutic targets based on the molecular signature of patient-derived xenografts in gastric cancer. J Hematol Oncol.

[22] Chen Z, Liu Z, Huang W, Li Z, Zou J, Wang J (2017). Gimatecan exerts potent antitumor activity against gastric cancer in vitro and in vivo via AKT and MAPK signaling pathways. J Transl Med.

[23] Wang J, Liu Z, Wang Z, Wang S, Chen Z, Li Z (2018). Targeting c-Myc: JQ1 as a promising option for c-Myc-amplified esophageal squamous cell carcinoma. Cancer Lett.

[24] Vorreuther R, Hake R, Borchmann P, Lukowsky S, Thiele J, Engelmann U (1997). Expression of immunohistochemical markers (PCNA, Ki-67, 486p and p53) on paraffin sections and their relation to the recurrence rate of superficial bladder tumors. Urol Int.

[25] Ichihara E, Westover D, Meador CB, Yan Y, Bauer JA, Lu P (2017). SFK/FAK signaling attenuates osimertinib efficacy in both drug-sensitive and drug-resistant models of EGFR-mutant lung cancer. Cancer Res.

[26] Zhang S, Yu D (2012). Targeting Src family kinases in anti-cancer therapies: turning promise into triumph. Trends Pharmacol Sci.

[27] Li D, Ambrogio L, Shimamura T, Kubo S, Takahashi M, Chirieac LR (2008). BIBW2992, an irreversible EGFR/HER2 inhibitor highly effective in preclinical lung cancer models. Oncogene.

[28] Cross DA, Ashton SE, Ghiorghiu S, Eberlein C, Nebhan CA, Spitzler PJ (2014). AZD9291, an irreversible EGFR TKI, overcomes T790M-mediated resistance to EGFR inhibitors in lung cancer. Cancer Discov.

[29] Soria J-C, Felip E, Cobo M, Lu S, Syrigos K, Lee KH (2015). Afatinib versus erlotinib as second-line treatment of patients with advanced squamous cell carcinoma of the lung (LUX-Lung 8): an open-label randomised controlled phase 3 trial. Lancet Oncol.

[30] Machiels J-PH, Haddad RI, Fayette J, Licitra LF, Tahara M, Vermorken JB (2015). Afatinib versus methotrexate as second-line treatment in patients with recurrent or metastatic squamous-cell carcinoma of the head and neck progressing on or after platinum-based therapy (LUX-Head & Neck 1): an open-label, randomised phase 3 trial. Lancet Oncol.

[31] Huang J, Fan Q, Lu P, Ying J, Ma C, Liu W (2016). Icotinib in patients with pretreated advanced esophageal squamous cell carcinoma with EGFR overexpression or EGFR gene amplification: a single-arm, multicenter phase 2 study. J Thoracic Oncol.

[32] Tajima Y, Shimada Y, Yagi R, Okamura T, Nakano M, Kameyama H (2016). A systematic analysis of oncogene and tumor suppressor genes for panitumumab-resistant rectal cancer with wild RAS gene - a case report. Gan To Kagaku Ryoho Cancer Chemother.

[33] Reckamp KL, Krysan K, Morrow JD, Milne GL, Newman RA, Tucker C (2006). A phase I trial to determine the optimal biological dose of celecoxib when combined with erlotinib in advanced non-small cell lung cancer. Clin Cancer Res.

[34] Banno E, Togashi Y, Nakamura Y, Chiba M, Kobayashi Y, Hayashi H (2016). Sensitivities to various epidermal growth factor receptor-tyrosine kinase inhibitors of uncommon epidermal growth factor receptor mutations L861Q and S768I: what is the optimal epidermal growth factor receptor-tyrosine kinase inhibitor?. Cancer Sci.

[35] Sequist LV, Waltman BA, Dias-Santagata D, Digumarthy S, Turke AB, Fidias P (2011). Genotypic and histological evolution of lung cancers acquiring resistance to EGFR inhibitors. Sci Transl Med.

[36] Rho JK, Choi YJ, Lee JK, Ryoo BY, Na II, Yang SH (2009). The role of MET activation in determining the sensitivity to epidermal growth factor receptor tyrosine kinase inhibitors. Mol Cancer Res.

[37] Kim LC, Song L, Haura EB (2009). Src kinases as therapeutic targets for cancer. Nat Rev Clin Oncol.

[38] Yoshida T, Zhang G, Smith MA, Lopez AS, Bai Y, Li J (2014). Tyrosine phosphoproteomics identifies both codrivers and cotargeting strategies for T790M-related EGFR-TKI resistance in non-small cell lung cancer. Clin Cancer Res.

