# Supplementary material for: Mouse avatar models of esophageal squamous cell carcinoma proved the potential for EGFR-TKI afatinib and uncovered Src family kinases involved in acquired resistance
Source: J Hematol Oncol. 2018 Aug 29;11:109. doi: 10.1186/s13045-018-0651-z (PMC6114252; doi:10.1186/s13045-018-0651-z)
Supplement: Supplementary file 1 — Materials and Methods. (DOCX 22 kb) [file 13045_2018_651_MOESM1_ESM.docx]

**Additional file 1: Materials and Methods**

**Antibodies**

The following antibodies were purchased from Cell Signaling Technology: EGFR (#4267), pEGFR Y1068 (#3777), MET(#8198), ERK (#4695), pERK T202/Y204 (#4370), AKT(#4691), pAKT S473 (#4060), pAKT T308 (#13038),S6 (#2217), pS6 S240/S244 (#4858), SFKs (#2108), pSFKs Y416 (#2101), E-cadherin (#3195), Vimentin (#5741), CCND1 (#2978), CDK4 (#12790), CDK6 (#3136), P21 (#2947), P27 (#3686), BCL2 (#15071), BAX (#5023), PARP (#9532), cleaved PARP (#5625), Capase8(#4790),cleaved Capase8(#9496), horseradish peroxidase (HRP)-conjugated anti-mouse (#7076), and HRP-conjugated anti-rabbit(#7074). The actin antibody (#014M4759) was purchased from Sigma-Aldrich. The Ki-67 antibody (#ZM-0167) was were purchased from ZSGB-BIO (China).

**Dissolution and administration methods of all the drugs for tumor xenograft studies**

The drugs were dissolved as follows: gefitinib was dissolved in 0.5% methyl cellulose (#M6385, Sigma-Aldrich) with 1% Tween 80 (#0442, AMRESCO); afatinib, crizotinib, and dasatinib were dissolved in water; and osimertinib was dissolved in 1% DMSO with 30% polyethylene glycol (PEG) 300 (#90878, AMRESCO). The following daily doses were given: gefitinib, 50 mg/kg; afatinib, 15 mg/kg; osimertinib, 15 mg/kg; crizotinib, 25 mg/kg; and dasatinib, 15 mg/kg. Dual treatment groups were dosed as follows: afatinib (15 mg/kg) plus crizotinib (25 mg/kg); afatinib (15 mg/kg) plus dasatinib (15 mg/kg). All inhibitors were given via oral gavage. Cetuximab and nimotuzumab were dosed at 0.5 mg per mouse by intraperitoneal injection (i.p.) twice a week.

**Next-generation panel sequencing and data analysis**

Briefly,~0.5 µg DNA per sample was used to generate capture-based sequencing libraries with a KAPA Hyper Prep Kit (Kapa Biosystems) followed by enrichment for 483 cancer-related genes using an Agilent SureSelect XT Target Enrichment System (Agilent Technologies) according to the manufacturer’s recommendations. The capture-based sequencing libraries were then sequenced on an Illumina Hiseq2000 system (Illumina) and 90–150 bp paired-end reads were generated.

After data filtration, high quality clean reads were aligned to the NCBI human reference genome (hg19) with BWA software, duplicates were marked with Picard software, and the alignment accuracy was improved with Genome Analysis Toolkit. Mutations, including single nucleotide variant (SNV) and InDel, were detected by Mutect and Strelka software, with further annotation by Annovar software. Copy number variations (CNV) were detected using the Event-wise testing algorithm as reported previously [[1](#_ENREF_1)].

**Transcriptome sequencing (RNA-seq) and data analysis**

Briefly, ~3 µg RNA per sample was used to generate sequencing libraries with NEBNext® Ultra™ RNA Library Prep Kit for Illumina® (NEB) according to manufacturer’s recommendations. Then, index codes were added to attribute sequences to each sample. Finally, the library preparations were sequenced on an Illumina Hiseq 2500 platform (Illumina) and 125–150 bp paired-end reads were generated.

The *homo sapiens* genome sequences and annotated gene models were downloaded from UCSC. STAR v2.5.1b was used to align the raw reads to genome sequences and HTSeq v0.6.1 was used to count the read numbers and calculate fragments per kilobase of transcript sequence per millions base pairs sequenced (FPKM) of each gene. For samples with biological replicates, differential expression analysis was performed using the DESeq2 R package (1.10.1); an adjusted P-value <0.05 was set as the threshold for significantly differential expression. For samples without biological replicates, differential expression analysis was performed using the edgeR R package (3.12.1), with a corrected P-value of 0.05 and an absolute fold change of two assigned as differentially expressed. GO and KEGG enrichment analyses were performed by the cluster Profiler R package. SNP calling was performed using GATK2 (v3.2.1) software followed by annotation for variable sites with SnpEff software.

**Reference**

1. Yoon S, Xuan Z, Makarov V, Ye K, Sebat J. Sensitive and accurate detection of copy number variants using read depth of coverage. Genome research. 2009;19(9):1586-92.
