# Supplementary material for: Mouse avatar models of esophageal squamous cell carcinoma proved the potential for EGFR-TKI afatinib and uncovered Src family kinases involved in acquired resistance
Source: J Hematol Oncol. 2018 Aug 29;11:109. doi: 10.1186/s13045-018-0651-z (PMC6114252; doi:10.1186/s13045-018-0651-z)
Supplement: Supplementary file 2 — Table S1. Efficacy of different EGFR blockers on ESCC cell lines. (DOCX 17 kb) [file 13045_2018_651_MOESM2_ESM.docx]

**Additional file 2: Table S1**

Efficacy of different EGFR blockers on ESCC cell lines.

|  |  |  |  |  |  |  |
| --- | --- | --- | --- | --- | --- | --- |
|  | IC50(μM) | | |  | IC50(μg/ml) | |
| Cell line | Gefitinib | Afatinib | Osimertinib |  | Cetuximab | Nimotuzumab |
| EC109 | 2.214±0.1255 | 0.1365±0.054615 | 0.379±0.0286 |  | >1000 | >1000 |
| KYSE450 | 1.357±0.2975 | 0.2322±0.0367 | 0.6769±0.10305 |  | >1000 | >1000 |
| KYSE140 | 0.3125±0.04965 | 0.02358+0.007675 | 0.1829±0.0197 |  | 712.3±288.05 | >1000 |
| KYSE510 | 7.809±1.1175 | 6.652±1.7515 | 1.742±0.298 |  | >1000 | >1000 |
| TE-1 | 5.072±1.5065 | 1.427±0.2875 | 2.498±0.3535 |  | 318.1±108.15 | >1000 |
| TE-10 | 10.887±1.435 | 1.605±0.292 | 2.575±0.5565 |  | >1000 | >1000 |
|  |  |  |  |  |  |  |
|  |  |  |  |  |  |  |

NOTE: Shown are the IC50 values of different EGFR blockers using CCK-8 assay, as discribed in

Materials and Methods.
