# Supplementary figures and images for: Mouse avatar models of esophageal squamous cell carcinoma proved the potential for EGFR-TKI afatinib and uncovered Src family kinases involved in acquired resistance
Source: J Hematol Oncol. 2018 Aug 29;11:109. doi: 10.1186/s13045-018-0651-z (PMC6114252; doi:10.1186/s13045-018-0651-z)

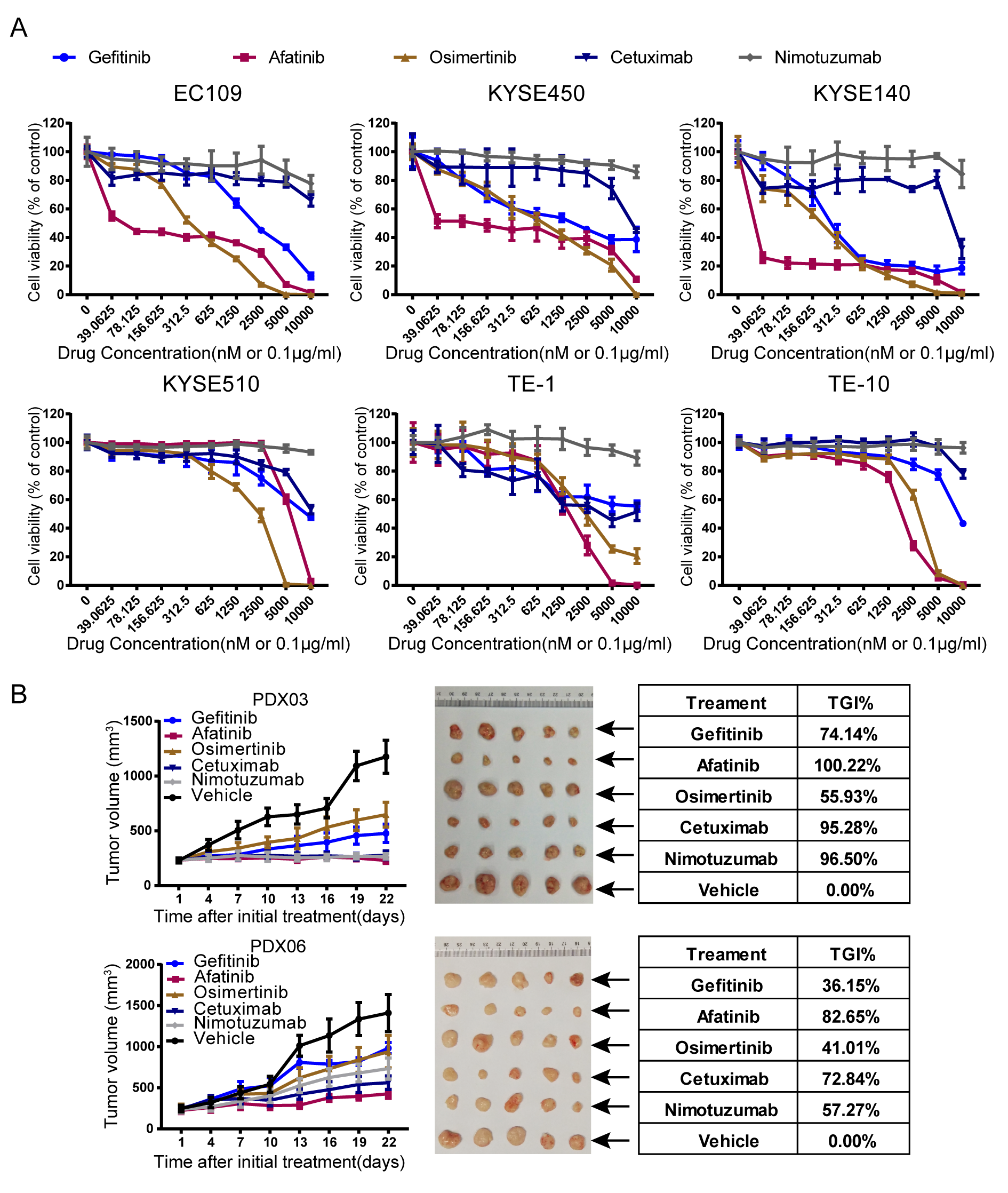

Supplement: Supplementary file 3 — Figure S1. Efficacy of different EGFR blockers on ESCC cell lines and PDX models. (A) Six ESCC cell lines were treated with different EGFR blockers (gefitinib, afatinib, osimertinib, cetuximab, and nimotuzumab) at the indicated concentrations (from 0 to 10 μM for TKIs and 0–1000 μg/mL for mAbs). After treatment for 72 h, cell growth inhibition was detected using CCK-8 assays. Cell viability at different doses relative to vehicle-treated controls is shown (means ± SD; three independent assays). (B) Curves plots the growth of PDX03 and PDX06 treated with vehicle control, gefitinib (50 mg/kg/day, oral gavage), afatinib (15 mg/kg/day, oral gavage), osimertinib (15 mg/kg/day, oral gavage), cetuximab (0.5 mg per mouse, twice a week, i.p.), or nimotuzumab (0.5 mg per mouse, twice a week, i.p.). Mice were sacrificed after 21 days of treatment and xenografts were isolated. Pictures of the xenografts are shown and the corresponding TGI is listed in the tables. Data are presented as means ± SDs; n = 5. (DOCX 1320 kb) [file 13045_2018_651_MOESM3_ESM.docx]

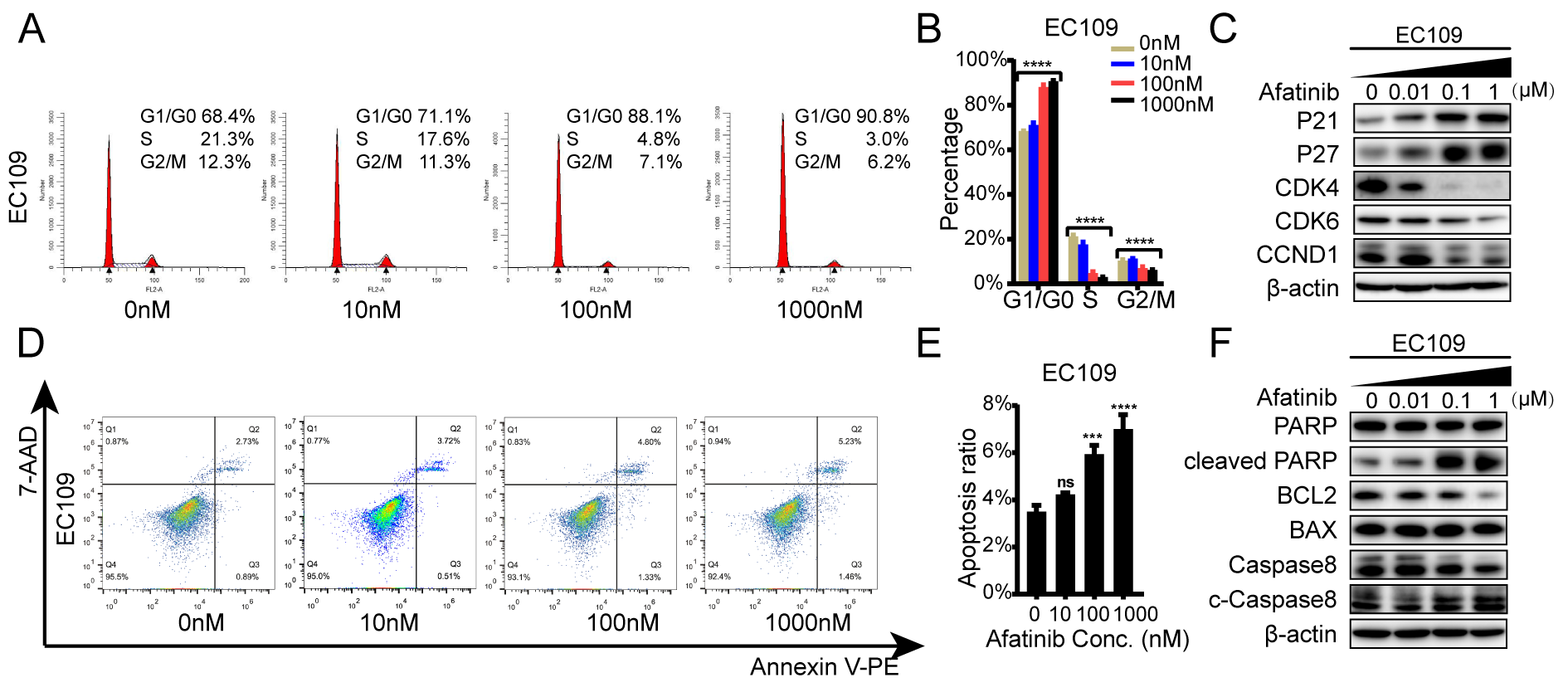

Supplement: Supplementary file 4 — Figure S2. Effects of afatinib on cell cycle and apoptosis in EC109 cells. EC109 cells were treated with 0, 10 nM, 100 nM, and 1 μM afatinib after serum-starvation for 12 h. After 48 h treatment, the cells were harvested and assayed as described below. The effects of afatinib on cell cycle distribution were assessed using flow cytometry after PI/RNase staining (A). The distribution of cells in the cell cycle is depicted (B). G1 phase-associated proteins (P21, P27, CDK4, CDK6, and CCND1) were assessed using western blotting (C).Flow cytometry showed the apoptosis induced by afatinib treatment using PE-annexin V and 7-AAD staining (D). The percentage of cells in early apoptosis (Q3) and late apoptosis (Q2) was calculated as the total apoptosis ratio (E). Apoptosis-related proteins (c-PARP, c-caspase8, BCL2, and BAX) were measured by western blotting after afatinib treatment (F). Data are presented as means ± SDs of three independent assays. P values were calculated using one-way ANOVA or unpaired two-tailed t-tests.*P < 0.05; **P < 0.01; ***P < 0.001; ****P < 0.0001; ns = not significant. (DOCX 570 kb) [file 13045_2018_651_MOESM4_ESM.docx]

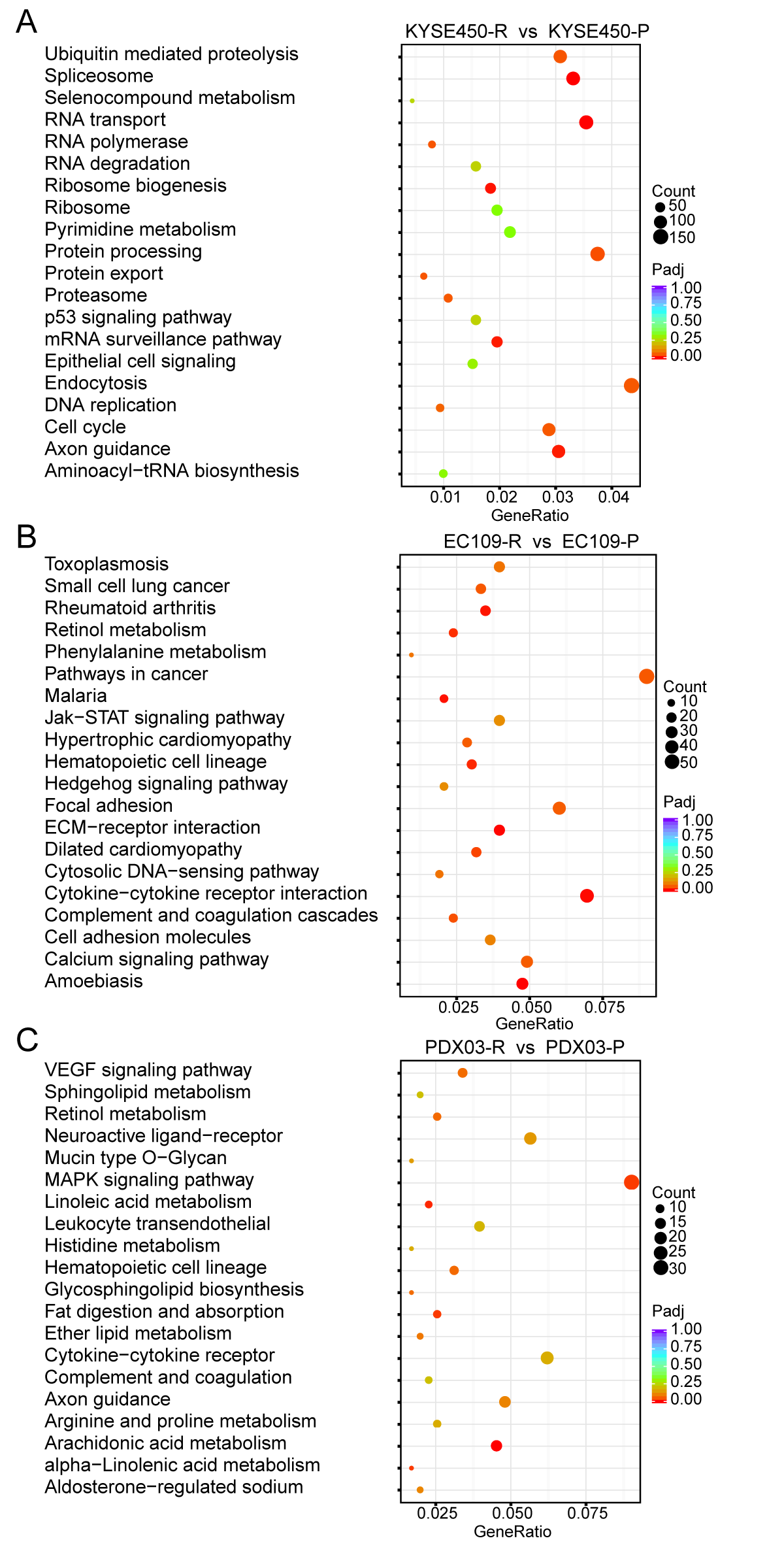

Supplement: Supplementary file 5 — Figure S3. Pathway enrichment by RNA-Seq. Dot plots showing the enrichment results of KEGG pathway analysis for KYSE450-R versus KYSE450-P (A), EC109-R versus EC109-P (B), and PDX03-R versus PDX03-P (C), as detected by RNA-seq. The size of the dots indicates the number of genes enriched in the corresponding pathways. The color of the dots indicates the significance level of the enriched pathways, as represented by the value of Padj. (DOCX 549 kb) [file 13045_2018_651_MOESM5_ESM.docx]
